# Bodipy Derivatives as Triplet Photosensitizers and the Related Intersystem Crossing Mechanisms

**DOI:** 10.3389/fchem.2019.00821

**Published:** 2019-12-12

**Authors:** Kepeng Chen, Yu Dong, Xiaoyu Zhao, Muhammad Imran, Geliang Tang, Jianzhang Zhao, Qingyun Liu

**Affiliations:** ^1^State Key Laboratory of Fine Chemicals, School of Chemical Engineering, Dalian University of Technology, Dalian, China; ^2^Key Laboratory of Energy Materials Chemistry, School of Chemistry and Chemical Engineering, Institute of Applied Chemistry, Xinjiang University, Ürümqi, China; ^3^College of Chemical and Environmental Engineering, Shandong University of Science and Technology, Qingdao, China

**Keywords:** Bodipy, intersystem crossing, photocatalysis, triplet photosensitizers, upconversion

## Abstract

Recently varieties of Bodipy derivatives showing intersystem crossing (ISC) have been reported as triplet photosensitizers, and the application of these compounds in photocatalysis, photodynamic therapy (PDT), and photon upconversion are promising. In this review we summarized the recent development in the area of Bodipy-derived triplet photosensitizers and discussed the molecular structural factors that enhance the ISC ability. The compounds are introduced based on their ISC mechanisms, which include the heavy atom effect, exciton coupling, charge recombination (CR)-induced ISC, using a spin converter and radical enhanced ISC. Some transition metal complexes containing Bodipy chromophores are also discussed. The applications of these new triplet photosensitizers in photodynamic therapy, photocatalysis, and photon upconversion are briefly commented on. We believe the study of new triplet photosensitizers and the application of these novel materials in the abovementioned areas will be blooming.

## Introduction

Triplet Photosensitizers (PSs) are compounds showing strong absorption of UV or visible light, efficient intersystem crossing (ISC), appropriate excited state redox potential, and long triplet-state lifetimes. These compounds are widely used for photo-driven energy transfer or electron transfer processes, which are the fundamental photophysical processes in photocatalysis, such as catalytic H_2_ evolution by water splitting (DiSalle and Bernhard, 2011; Gärtner et al., 2011, 2012), photocatalytic redox synthetic organic reactions (Shi and Xia, 2012; Xuan and Xiao, 2012; Hari and König, 2013), photoreduction of CO_2_ (Sato et al., 2013), photodynamic therapy (PDT) (Awuah and You, 2012; Kamkaew et al., 2013; Stacey and Pope, 2013; Jiang et al., 2016; Li et al., 2017), photon upconversion (triplet-triplet annihilation upconversion) (Singh-Rachford and Castellano, 2010; Ceroni, 2011; Zhao et al., 2011; Monguzzi et al., 2012; Simon and Weder, 2012; Zhou et al., 2015), photovoltaics (Guo et al., 2006; Dai et al., 2012; Bittner et al., 2014; Cheema et al., 2014; Etzold et al., 2015), and photo-initiated polymerizations (Goessl et al., 2001; Ho et al., 2010; Rivard, 2012; Cengiz et al., 2017). It is highly desired to find a chromophore to develop a series of triplet PSs to meet these requirements. Concerning this aspect, Boron dipyrromethene (Bodipy) is of particular interest due to its robust photostability and feasible derivatization (Ulrich et al., 2008; Ziessel and Harriman, 2011; Lu et al., 2014; Miao et al., 2019).

The ISC process is electron spin forbidden, and a mechanism is thus required to enhance the electron spin flipping, which requires a magnetic torque. Recently, a variety of Bodipy-based triplet PSs have been reported, and the application of these novel triplet PSs in the abovementioned areas is promising. In this review, we have summarized the recent development of the Bodipy-derived triplet PSs, ranging from the molecular structure design to the applications of these materials.

S_n_ → T_m_ ISC is a non-radiative transition, during which the electron spin flips or rephrases. A magnetic torque acting on the electron spin is therefore required. The most commonly encountered mechanisms are the spin-orbital coupling (SOC) and hyperfine interaction. For the SOC, the orbital angular momentum interacts with the spin angular momentum (also known as the magnetic angular momentum interaction), and the typical examples are the heavy atom effect, the El Syed's rule (nπ^*^ ↔ ππ^*^ transition), the spin orbital charge transfer, etc. Hyperfine interaction refers to the magnetic coupling between the electron and the magnetic nucleus, which is responsible for the radical pair ISC (RP ISC) in electron donor/acceptor dyads. Other ISC mechanisms do exist; for instance, the exciton coupling and singlet fission. In the following sections we have introduced the exemplars of application of Bodipy derivatives as triplet photosensitizers (Lakshmi et al., 2015). The challenge in the designing of heavy atom-free triplet photosensitizers were also introduced.

## Heavy Atom Effect in Bodipy Derivatives

Nagano et al. reported that the 2,6-diiodoBodipy (Supplementary Figure 1) showed the ISC ability (Yogo et al., 2005). The ISC was confirmed with the photosensitizing of singlet oxygen (confirmed with the near IR luminescence of ^1^O_2_), and the relative efficiency of ^1^O_2_ generation is up to 6-fold of that of Rose Bengle under the same conditions. The photostability of the diiodoBodipy is better than the Rose Bengle. Intracellular phototoxicity was also confirmed. However, the triplet-state lifetime of the diiodoBodipy was not reported.

**Figure 1:**
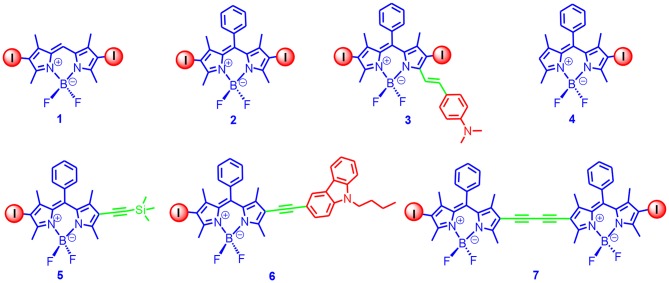
Mono- and DiiodoBodipy derivatives showing variable absorption wavelengths and efficient ISC.

Based on the feasible derivatization of the Bodipy framework, we prepared a library of iodinated Bodipy derivatives (Figure 1); the absorption wavelength ranged from 510 to 629 nm, and the T_1_ state energy level of the derivatives varied from 1.5 to 1.15 eV (based on TDDFT computations) (Wu et al., 2011). The visible light absorbing of these derivatives was strong (59,400–180,000 M^−1^ cm^−1^). With nanosecond transient absorption spectra of the diiodoBodipy derivatives, the triplet-state lifetimes of the compounds were determined to be in the range of 26–66 μs. It is worth mentioning that these *apparent* triplet-state lifetimes were shorter than the *intrinsic* triplet-state lifetimes as a result of the triplet-triplet-annihilation (TTA) self-quenching effect. Later, Zhao and Dick reported a kinetic model with the TTA effect that was considered to determine the intrinsic triplet-state lifetimes, and the triplet-state lifetime of the diiodoBodipy was up to 276 μs (Lou et al., 2018; Wang et al., 2019b). To the best of our knowledge, this was the first report of the intrinsic triplet-state lifetime of Bodipy derivatives.

Since these diiodoBodipy derivatives showed strong absorption of visible light, an efficient ISC, and a long-lived triplet state, we used these triplet PSs for TTA upconversion (Wu et al., 2011). For instance, upon 532 nm cw-laser excitation, strong upconverted blue emission of ca. 450 nm was observed with perylene as the triplet acceptor/emitter, and the upconversion quantum yields were up to 6% (Wu et al., 2011). Other excitation wavelengths can be also used for TTA upconversion with these compounds.

In Figure 1, the methyl groups at the 1,7-position of the Bodipy core restrict the rotation of the phenyl ring at the meso-position. Without the iodination and the methyl groups at the 1,7-positions, the rotation of the phenyl ring will induce the free rotor effect, and the fluorescence of the Bodipy core is significantly quenched. A fluorescence sensor for detection of the viscosity of the microenvironment has been developed based on this property because the rotation of the phenyl ring will be inhibited in a viscous solvent (Kuimova et al., 2008). With theoretical computation, it was proposed that the quenching of the fluorescence of the Bodipy core was not due to rotation of the phenyl ring at the meso-positions, but rather that it was the bending of the Bodipy core at the excited state that quenched the fluorescence (Suhina et al., 2017).

**Figure 2:**
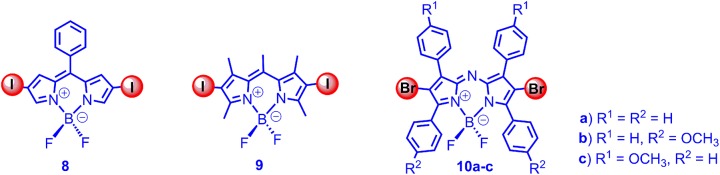
DiiodoBodipy derivatives (8, 9), which are devoid of free rotor effect on the triplet states and dibromoAzaBodipy (10a-c), showing absorption at 650–690 nm.

With the initial intention to develop a viscosity-sensitive triplet photosensitizer, we studied the triplet-state property of the diiodoBodipy without methyl groups at 1,7-position (**8**, **9** in Figure 2). Interestingly, we found that the triplet state in **8** was not quenched, as compared to that of **9**, in both aspects of triplet-state lifetimes (126 μs for **8**, 241 μs for **9**) and ISC quantum yields (approximated with the O.D. values of the nanosecond transient absorption) (Lou et al., 2018). The non-quenched triplet state of **8** was also confirmed with the TTA upconversion with this triplet PS (upconversion quantum yield was up to 6.3%).

With theoretical computation, we found that, with torsion of the molecule at excited state, there is a crossing for S_1_/S_0_ at the energy minimum on the S_1_ state energy curve. For T_1_ state, however, there is not such crossing point for the potential energy curves (Lou et al., 2018), which can be used to rationalize the un-quenched T_1_ state of **8** and the quenched S_1_ state of the same molecule. The electron spin-forbidden feature of the T_1_ → S_0_ may also play a role in the observation of the non-quenched T_1_ state of **8**. These results indicated the triplet-state property was different from the singlet excited state property for the same molecule.

While the normal Bodipy showed absorption in the green range (ca. 500 nm), the azaBodipy showed much red-shifted absorption in the range of 680 nm. O'shea et al. prepared dibromoazaBodipy (**10** in Figure 2), and the compounds showed strong absorption in the near IR spectral region (the molar absorption coefficients were up to 80,000 M^−1^ cm^−1^) (Gorman et al., 2004; Palma et al., 2009). The fluorescence quantum yields of the dibromoazaBodipy were low (1–10%). The ISC ability of the compounds were confirmed by singlet oxygen photosensitizing, and the PDT effect was studied with cancer cells (Gorman et al., 2004). However, the triplet-state lifetimes of the compounds were not reported.

**Figure 3:**
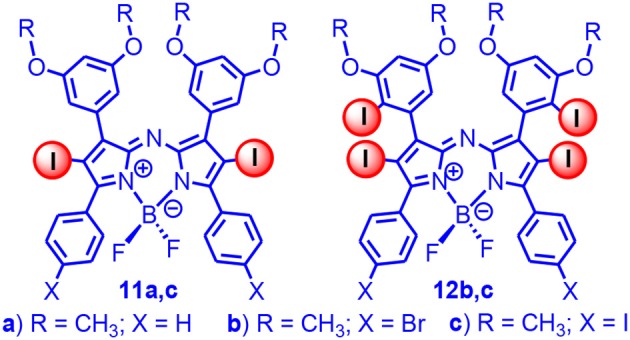
Di- and tetraiodoAzaBodipy showing absorption at 650–690 nm.

Ramaiah et al. prepared a series of iodinated analogs (Figure 3; Adarsh et al., 2012), and the absorption wavelength and the absorptivity were similar to the brominated analogs. With nanosecond transient absorption spectra, the triplet-state lifetimes of the diiodoazaBodipys were determined as *ca*. 2 μs, and triplet-state quantum yields were determined as 70–80% (Adarsh et al., 2012). Besides the azaBodipy derivatives, the styryl Bodipy also showed absorption in the red spectral region (Deniz et al., 2008; Lu et al., 2014). We studied the triplet-state property of the 2,6-diiodobisstyrylBodipy; the triplet-state lifetime was determined as 1.8 μs, and the singlet oxygen quantum yield was determined as 69% (Huang et al., 2013b). Notably, the triplet-state lifetime was much shorter than the normal diiodoBodipy (*ca*. 270 μs). We proposed that the energy gap law alone is not the reason for the short triplet-state lifetimes (Liu and Zhao, 2012; Sun et al., 2013b; Yang et al., 2016). To the best of our knowledge, this was the first time that the triplet-state property of the styryl substituted Bodipy was studied. We also used the 2,6-diiodoBodipy as a novel photocatalyst for an Aza Henry reaction (oxidative coupling of benzylamine); the reaction was more efficient than the conventional photocatalysts, such as the Ru(bpy)_3_ or Ir(ppy)_3_ (Huang et al., 2013b). We attributed the efficient photocatalysis to the strong absorption of visible light by the diiodostyrylBodipy.

The heavy atom effect for enhancing spin–orbit coupling is a common mechanism to facilitate the ISC of Bodipy compounds (Supplementary Figure 2; Wu et al., 2011; Chen et al., 2012; Nakashima et al., 2018). Furthermore, Eisenberg and MaCamant studied the ISC kinetics of the 2,6-dibromoBodipy and the 2,6-diiodoBodipy (Sabatini et al., 2011). Based on the decay of the stimulated emission (SE) band, the ISC time constants of the 2,6-dibromoBodipy and the 2,6-diiodoBodipy were determined as 1.3 ns and 127 ps, respectively. This result indicated that the iodine atom was more efficient in inducing ISC. It is worth mentioning that the heavy atoms should be attached to the π-conjugation framework of the Bodipy, not to the peripheral moieties, as otherwise the heavy atom effect-induced ISC would not be efficient (Singh-Rachford et al., 2008).

## Transition Metal Complexes Containing Bodipy Moieties

Transient metal complexes normally show partially forbidden S_0_ → ^1^MLCT transition; as a result, the absorption in the visible range is normally weak. Moreover, because of the significant involvement of the transition metal in the transitions, the T_1_ state is actually strongly quenched, manifested by its short triplet-state lifetime (Williams, 2007; Wong and Yam, 2007). Incorporation of a visible light-harvesting chromophore in the transition metal complex will have at least two possible benefits: to strengthen the visible light harvesting and to prolong the triplet-state lifetimes. The achievement of these goals, however, is dependent on the molecular structure design.

**Figure 4:**
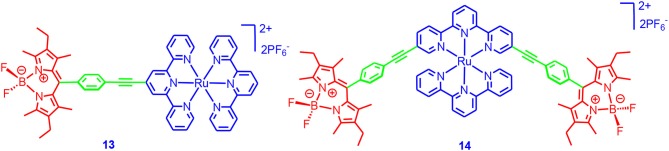
Ru(II) complex containing Bodipy appendants.

Campagna and Ziessel et al. reported Ru(II) terpyridine complexes containing a Bodipy chromophore (Figure 4; Galletta et al., 2005). A population of the Bodipy-localized triplet state (lifetimes are 8 and 30 μs, respectively) were observed for both complexes. Phosphorescence of the ^3*^Bodipy was observed at 77 K. However, we hypothesized that the ISC, upon excitation of the Bodipy unit, was probably not efficient due to the large distance between the Ru(II) center and the Bodipy moiety. It should be noted that the triplet-state lifetime of the parent Ru(II) (terpyridine)_2_ was short (250 ps), and the long triplet-state lifetimes of **13** and **14** were due to the energy transfer and the localization of triplet state on the Bodipy part. This is a typical example for manipulation of the triplet-state property of transition metal complexes with ligand modification (McClenaghan et al., 2005; Campagna et al., 2007).

**Figure 5:**
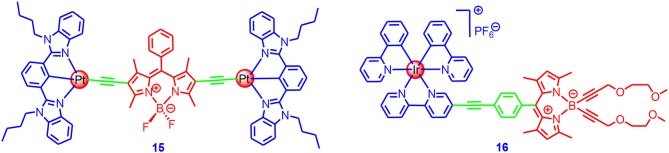
N^∧^C^∧^N Pt(II)–acetylide complex and Ir(III)(ppy)(bpy) complex contains Bodipy chromophore.

Inspired by the molecular structures in the literature (Nastasi et al., 2008), we theorized that directly linking the π-conjugation framework to a metal center may greatly enhance the ISC of the chromophore as this will ensure the excitation energy harvested by the complexes will be efficiently transformed into triplet-state energy (**15** in Figure 5; Wu et al., 2012a). Complex **15** was based on a N^∧^C^∧^N Pt(II)–acetylide coordination profile, which showed high phosphorescence quantum yields (19%) (Tam et al., 2011). The absorption of **15** was redshifted (absorption maximum was at 574 nm), as compared to the free Bodipy ligands (absorption band was centered at 543 nm), indicating strong electronic interaction between the Pt(II) coordination center and the Bodipy chromophore. Room temperature near IR phosphorescence band centered at 770 nm was observed, and the phosphorescence quantum yield was up to 3.5%. The triplet-state lifetime was determined as 128 μs by nanosecond transient absorption spectroscopy (Wu et al., 2012a). To the best of our knowledge, this was the first report of the strong near-IR phosphorescence of Bodipy at room temperature. This property indicated the efficiency of the ISC. The complex was used as a triplet photosensitizer for TTA upconversion, and upconversion of the quantum yield up to 5.2% was observed.

Bodipy chromophores were also attached to Ir(III) coordination frameworks. Castellano and Ziessel prepared complex **16**, in which the Bodipy chromophore was attached to the Ir(III) coordination center via an ethynyl linker at the meso-phenyl moiety (**16** in Figure 5; Rachford et al., 2010). The complex showed a strong absorption band centered at 501 nm, and it showed the same for the Bodipy ligand, indicating limited electronic interaction between the Ir(III) coordination center and the Bodipy chromophore at the ground state. This is also supported by the redox potentials of the complex and the free ligands. Nanosecond transient absorption spectroscopy showed the Bodipy-localized triplet state with a lifetime of 25 μs. The residual fluorescence of the Bodipy ligand was observed for the complex, and a phosphorescence band centered at 730 nm was observed at 77 K.

**Figure 6:**
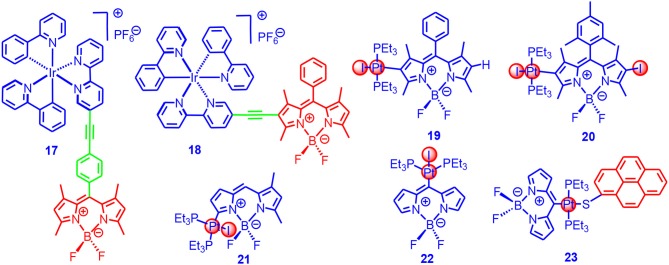
Ir(III)(ppy)(bpy) complex (17, 18) contains Bodipy chromophores via conjugated and non-conjugated linkers and σ-Pt-BODIPY complexes (19–23) with the Bodipy carbon directly metalated.

We studied the effect of π-conjugated and non-conjugated linkers on the photophysical properties of the Ir(III) complexes (**17**, **18** in Figure 6; Sun et al., 2013a). The molecular structure of **17** was similar to complex **16**. In complex **18**, however, the π-conjugated framework of the Bodipy chromophore was connected to the Ir(III) coordination center, which was different from that in **17**. Although **18** showed the same absorption wavelength as compared to the free ligand, room temperature phosphorescence at 742 nm was observed (phosphorescence quantum yield: 0.03%), although with the residual fluorescence of the Bodipy unit at 553 nm (yield 0.3%). We noted the residual fluorescence of the Bodipy unit in **17** was higher (1.8%), and no phosphorescence was observed for **17**. These results indicated the ISC in **18** was more efficient than **17**. This conclusion was supported by the singlet oxygen photosensitizing studies; for **18**, the singlet oxygen quantum yield (Φ_Δ_) was 97%, whereas, for **17**, the Φ_Δ_ was 52%. These results demonstrated that the structure of the linker must be taken into account in order to ensure an efficient ISC.

The triplet-state lifetimes of **17** and **18** were determined as 23.7 and 87.2 μs, respectively. We used the photosensitizer for photocatalytic oxidation of 1,5-dihydroxylnaphthalene, and results showed that the Ir(III) complexes containing the Bodipy units were much more efficient than the conventional Ir(ppy)_2_bpy complex. The complexes were also used as triplet photosensitizers for TTA upconversion, with perylene as the triplet acceptor/emitter. The TTA upconversion efficiency with **18** was 2.8%, whereas it was 1.2% for **17**. The parent complex Ir(ppy)_2_bpy showed an upconversion quantum yield of 0.3%. We expect that the transition metal complexes showing strong absorption of visible light and a long-lived triplet state will be promising for applications in photocatalysis (Sun et al., 2012, 2013b; Guo et al., 2018; Wang et al., 2019a).

Winter et al. directly attached the Bodipy chromophore to the Pt (**19**-**23** in Figure 6) without the ethyne linker, and thus the ISC was supposed to be maximized for these so-called σ-Pt-BODIPY complexes (Irmler and Winter, 2016, 2018; Irmler et al., 2019a,b). The photophysical properties, as well as the electrochemical properties, are substitution position dependent. Dual emission bands centered at 588 and 797 nm were observed for the complexes, with the phosphorescence quantum yield up to 0.364%. The singlet oxygen quantum yields were *ca*. 50% for the complexes with metalation at the 2- or 3-positions, and it reached 95% for the complex with metalation at the 8-position (Irmler and Winter, 2018). Complexes containing both Bodipy and pyrene ligands (**23**) were prepared (Irmler and Winter, 2016; Irmler et al., 2019a,b), and charge separation (CS) and energy transfer were observed with the complexes. The CT phosphorescence emission band was at 724 nm, and the singlet and triplet emissions of the Bodipy-localized excited state were at 470 and 635 nm, respectively. The phosphorescence lifetimes were up to 500 μs. Interestingly, the complexes with 8-metalation show blueshifted emission as compared to the complexes with metalation at 2-positions (Leen et al., 2012). The ISC mechanism of σ-Pt-BODIPY complexes is presented in Supplementary Figure 3.

## Orthogonal Bodipy Dimers: the ISC Mechanism

Previously, some Bodipy dimers were reported to show the exciton coupling effect (Supplementary Figure 4; Bröring et al., 2008; Ventura et al., 2009), which requires specific orientation of the two chromophores (Kasha et al., 1965). These compounds have been revised recently, and readers are suggested to refer those review articles (Zhao et al., 2013, 2015). The manifestation of the exciton coupling is normally a significant splitting of the absorption band of the monomer chromophore in the dimers or dyads (Bröring et al., 2008; Ventura et al., 2009). In this case, one singlet state may share similar energy levels with a triplet state, and thus the ISC will be enhanced.

**Figure 7:**
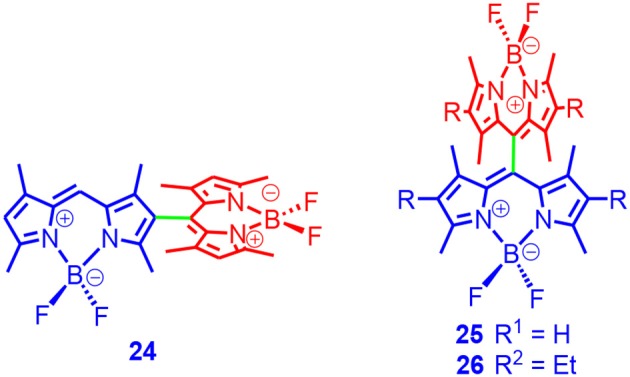
Orthogonal Bodipy dimers showing efficient ISC.

Recently, orthogonal Bodipy dimers were reported (dimers **24**, **25**, and **26** in Figure 7; Cakmak et al., 2011). In this case, there is no splitting of the absorption band, which is different from the exciton coupling effect. The absorption maxima of the compounds are close to the absorption of the monomer. The fluorescence quantum yields of dimers **24**, **25**, and **26** were 3, 31, and 49%, respectively. The singlet oxygen quantum yields of the compounds were 51, 46, and 21%, respectively. Phototoxicity was confirmed with cancer cells. However, the triplet-state property, such as the triplet-state lifetimes, were not studied. Later, Akkya and Dede et al. proposed that the ISC was due to a doubly excited state mechanism (Duman et al., 2012).

**Figure 8:**
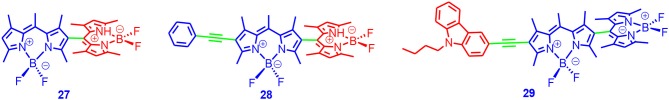
Orthogonal Bodipy dimer 27 and heteroBodipy dimers 28 and 29 showing efficient ISC.

We prepared an orthogonal Bodipy dimer **27**, and two hetero-Bodipy dimers, **28** and **29**. The aim was to extend the absorption wavelength (Figure 8; Wu et al., 2013). Dimer **27** showed one major absorption band centered at 506 nm, indicating that the two subunits in **27** were identical. For **28** and **29**, however, two absorption bands centered at 509 and 541 nm were observed, which indicated that the two parts were not identical. The fluorescence quantum yields of **27, 28**, and **29** were determined as 2.2, 17.6, and 2.3%, respectively (in DCM). The Φ_Δ_ values were 64 and 42% for **27** and **28**; no singlet oxygen production was observed for **29**. With nanosecond transient absorption spectroscopy, we determined the triplet-state lifetime of **27** and **28** as 115.6 and 140.9 μs, respectively. Note these apparent triplet-state lifetimes were shorter than the intrinsic triplet-state lifetimes as a result of TTA quenching effect. To the best of our knowledge, this is the first time that the triplet excited state of Bodipy dimers was reported. We proposed that the heavy atom-free triplet photosensitizers were superior, and that the triplet-state lifetime was long, which made it beneficial for intermolecular electron transfer or the charge transfer processes. Thus, we used the dimers as triplet photosensitizers for TTA upconversion, and perylene was used as triplet acceptor/emitter. The upconversion quantum yield was determined as 3.7%. This example demonstrated that the heavy atom-free triplet photosensitizer based on the Bodipy dimers were applicable to intermolecular triplet energy transfers like TTA upconversion.

The ISC mechanisms of these orthogonal Bodipy dimers are controversial. Initially, it was proposed the doubly excited state was responsible for the ISC (Duman et al., 2012). Later, it was proposed that charge transfers between the two units are involved, and that the charge recombination induced the ISC. This was based on an observation of the charge transfer state with the femtosecond transient absorption spectroscopy (Epelde-Elezcano et al., 2017; Liu et al., 2018). It was proposed that the spin–orbital charge-transfer ISC (SOCT-ISC) was responsible for the ISC. Recently, it was proposed the ISC of these orthogonal Bodipy dimers was due to singlet fission (Montero et al., 2018). However, the time-resolved electron paramagnetic resonance (TREPR) spectroscopy study of the dimers does not support this postulation because no quartet state was observed (the spin–spin interaction was as a result of the triplet–triplet pair) (Kandrashkin et al., 2019).

Moreover, the donor/acceptor dyads with large separation distances between the electron donor and acceptor have been studied for a long time. The radical pair ISC (RP ISC) was found to be responsible for the ISC (Wiederrecht et al., 2000; Dance et al., 2006; Kc et al., 2014). However, the synthesis of these electron donor/acceptor dyads is difficult because of the rigid and long linkers in these compounds. Interestingly, some electron donor/acceptor dyads with simple molecular structures were reported recently to show ISC ability; these compounds are promising candidates for heavy atom-free triplet photosensitizers.

**Figure 9:**
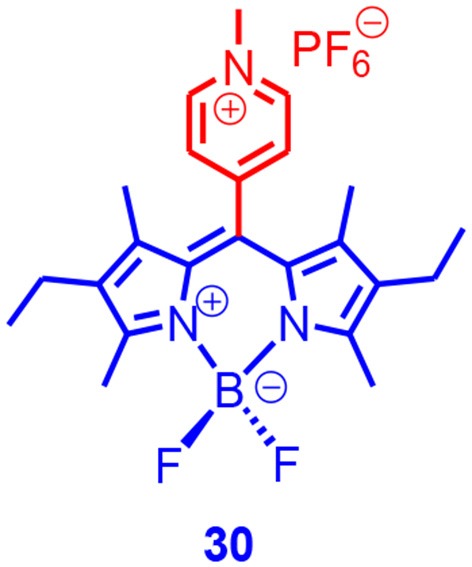
Bodipy derivative with pyridium electron acceptor unit.

Harriman and Ziessel reported a Bodipy derivative with pyridium moiety at the meso position (Figure 9). For the analog containing a neutral pyridyl moiety, the fluorescence quantum yield was high (78%) and the formation of triplet state was negligible (Harriman et al., 2007).

For compound **30**, however, the fluorescence quantum yield decreased to 0.5%, triplet-state formation upon photoexcitation was conformed with nanosecond transient absorption spectroscopy, the ISC quantum yield was determined as 75%, and the triplet-state lifetime was determined as 2.0 ± 0.5 μs. Based on picosecond transient absorption spectroscopy, the charge separation process had a time constant of 5 ps, and the charge recombination (CR)-induced ISC had a time constant of 0.7 ns (Harriman et al., 2007). The ISC should belong to the SOCT-ISC mechanism (Supplementary Figure 5) of the compact electron donor/acceptor dyads; the electron donor and acceptor should adopt orthogonal geometry, and the angular momentum conservation would thus be satisfied for the ISC (van Willigen et al., 1996; Dance et al., 2008). Energy levels of the Bodipy dimers do not support the singlet fission mechanism (excitation energy is 2.44 eV, whereas the T_1_ state energy of the Bodipy chromophore is ca. 1.70 eV) (Rachford et al., 2010). It should be pointed out that some Bodipy dimers do not show any ISC (Liu et al., 2018), although the so-called symmetry-breaking charge transfer (SBCT) still occurs (Whited et al., 2012).

## Charge Recombination-Induced ISC in Bodipy Derivatives

Charge recombination (CR)-induced ISC in electron donor/acceptor dyads with large separation distance between the electron donor and acceptor has been studied for a long time. Radical pair ISC (RP ISC) has been found to be responsible for the ISC (Wiederrecht et al., 2000; Dance et al., 2006; Kc et al., 2014). However, the synthesis of these electron donor/acceptor dyads is difficult because of the rigid and long linkers in these compounds. Interestingly, some electron donor/acceptor dyads with simple molecular structures were reported recently to show ISC ability; these compounds are promising candidates for heavy atom-free triplet photosensitizers (Filatov et al., 2017; Hou et al., 2019).

**Figure 10:**
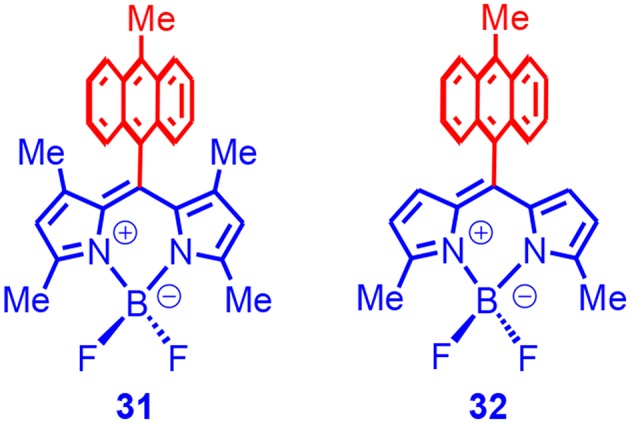
Bodipy derivative with anthryl moiety as electron donor.

Filatov and Senge reported feasibly prepared Bodipy–anthryl electron donor/acceptor dyads, in which the anthryl was used as an electron donor (Figure 10; Filatov et al., 2017). The fluorescence of the Bodipy moiety in the dyads was quenched to a large extent, and the CS in both dyads was confirmed with femtosecond transient absorption spectroscopy. The singlet oxygen quantum yields of the two dyads were determined as 67 and 38%, respectively. Among other factors, the orthogonal geometry in the dyad was beneficial for the higher singlet oxygen quantum yields. For **31**, the triplet-state lifetime was determined as 41 μs with nanosecond transient absorption spectroscopy. The same researchers prepared a series of analog Bodipy-derived electron donor/acceptor dyads. The dyads generally showed satisfactory SOCT-ISC (Filatov et al., 2018). Zhang also prepared Bodipy-based electron donor/acceptor dyads, and the SOCT-ISC was observed (Zhang and Feng, 2017; Zhang et al., 2017; Hu et al., 2019).

We prepared a series of Bodipy–anthryl dyads, in which the anthryl and the Bodipy units adopted orthogonal geometry, but the dipole moments of the two subunits were either in parallel or perpendicular (Figure 10; Wang and Zhao, 2017; Wang et al., 2019b), which was different from the previous reports of the anthryl–Bodipy dyads (Filatov et al., 2017).

**Figure 11:**
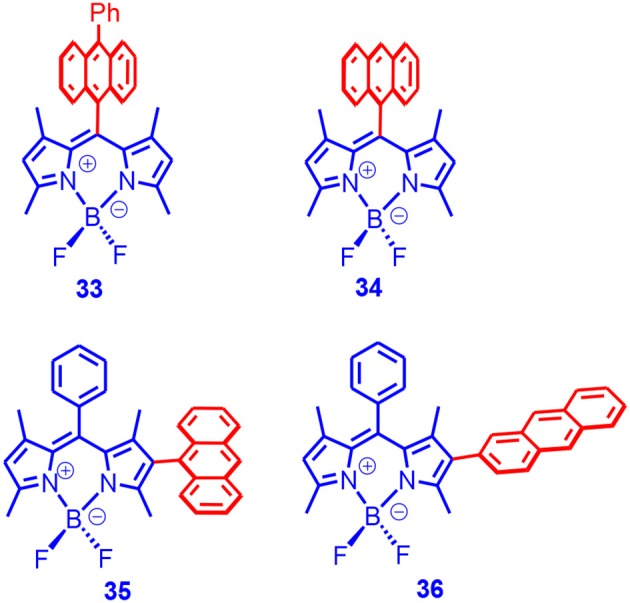
Bodipy derivative with anthryl moiety as electron donor and the dipole moments of the subunits are with parallel or perpendicular.

We found that, although the fluorescence of the Bodipy unit was all quenched in the dyads, the singlet oxygen quantum yield varied drastically. For **33** and **34**, the singlet oxygen quantum yield can be up to 90%, whereas for **35** and **36**, the singlet oxygen quantum yields were much lower (at most ca. 20% Figure 11). Delayed fluorescence was observed for the dyads (P-type, i.e., TTA mechanism). We observed a long triplet-state lifetime for the dyads (up to 82 μs). These dyads were used for TTA upconversion, and an upconversion quantum yield up to 15.8% was observed (Wang and Zhao, 2017). To the best of our knowledge, this was the first time that electron donor/acceptor dyads showing SOCT-ISC and strong absorption of visible light were used for TTA upconversion.

Moreover, we used TREPR spectroscopy to study the electron spin polarization (ESP) of the triplet state of the dyads. The purpose of this kind of study was to study the ISC mechanisms, e.g., to discriminate the radical pair ISC and the SOCT-ISC mechanisms. We observed an ESP of (*e, e, e, a, a, a*) for **33** and **34**, which was similar to that of 2,6-diiodoBodipy (Wang et al., 2019b). This finding was different from the previous reports that the ESP of the triplet state accessed with SOCT-ISC should be always different from the SO ISC (Dance et al., 2008). Interestingly, **35** and **36** showed an ESP of (*a, e, a, e, a, e*), which was different from that of **33** and **34**. The ESP ruled out the RP ISC mechanism. Interestingly, for **34**, three triplet states were simultaneously observed, i.e., the ^3^CT state, ^3^An state, and ^3^Bodipy states. It was proposed that the CR was inhibited to some extent at low temperatures. We have proposed that it is more convincing to use the potential energy curve of the torsion to study the molecular conformation rather than the single point optimization.

**Figure 12:**
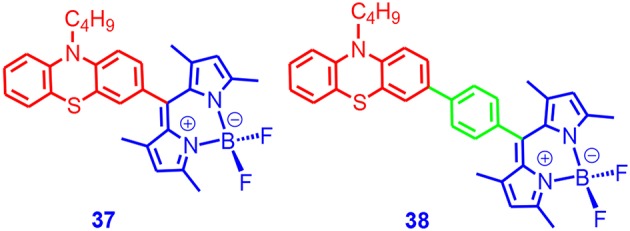
Bodipy derivative with phenothiazine (PTZ) moiety as electron donor.

We also used phenothiazine as a strong electron donor to construct SOCT-ISC dyads based on Bodipy (**37** and **38**, Figure 12; Chen et al., 2017). PTZ had an oxidation potential of +0.3 V (vs. Fc/Fc^+^), which was more negative than anthryl (ca. +1.0 V, vs. Fc/Fc^+^). One of the effects of using a stronger electron donor was the decreasing of the CT state energy levels, which may lead to changes of the energy level match profile between the CT state and the LE triplet state.

In other words, the solvent polarity dependency of the ISC quantum yields may change for **37** and **38**, as compared to that fluorescence. This was observed for the Bodipy–anthryl dyads (Filatov et al., 2017). The fluorescence of the Bodipy moiety in **37** (2.7%) was weaker than **38** (7.2%). Efficient singlet oxygen photosensitizing was observed for **37** (67% in toluene, much weaker in other solvents). The singlet oxygen photosensitizing of **38** was weaker (24.6%). Note for the Bodipy–anthryl dyads, high singlet oxygen photosensitizing was observed in acetonitrile (Wang et al., 2019b). An apparent triplet-state lifetime of 116 μs was observed with nanosecond transient absorption spectroscopy. We used **38** as triplet photosensitizer for TTA upconversion, and an upconversion quantum yield of 3.2% was observed.

## ISC of the C_60_-bodipy DYADS: C_60_ as the Electron Spin Converter for Achieving ISC

Fullerene C_60_ has been widely used as an electron acceptor in organic photovoltaics materials (Yamazaki et al., 2004; Chen et al., 2011; Izawa et al., 2011; Tamura et al., 2014). It was also used in electron transfer because of its small reorganization energy (Turro et al., 2009). However, we believe one of its photophysical properties has not been fully exploited, i.e., the efficient ISC (Arbogast et al., 1991). The ISC efficiency is close to a unit; however, C_60_ itself is not an ideal triplet photosensitizer because the absorption in the visible spectral region is very weak. We proposed that this drawback could be addressed by attaching a visible light-harvesting chromophore to C_60_. The energy transfer from the organic chromophore to the C_60_ would thus produce the S_1_ state of C_60_, then, via the ISC of the C_60_ unit, the triplet state would be populated. The final localization of the triplet state would be dependent on the relative triplet energy levels of the chromophore and the C_60_ unit. Moreover, charge separation cannot be excluded for the C_60_-Bodipy dyads, especially in highly polar solvents.

**Figure 13:**
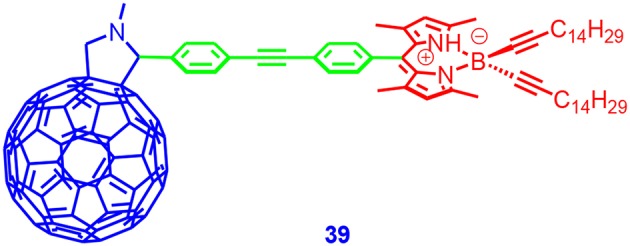
Bodipy–C_60_ dyad derivative with Bodipy moiety as electron donor.

Ziessel and Harriman prepared a C_60_-Bodipy dyad (**39** in Figure 13; Ziessel et al., 2009). The fluorescence of the Bodipy unit was strongly quenched in the dyad. Based on picosecond transient absorption as well as electrochemical studies, it was found the singlet energy transfer was dominant upon photoexcitation of the C_60_ unit in non-polar solvents. Finally, the triplet state on the C_60_ unit was populated. In polar solvent benzonitrile, the CT state contained lower energy than the ^3*^C_60_ state. The CT state had a lifetime of 430 ps. In DCM, the formation of the CT state was observed; the lifetime was 160 ps, the CR lead to the formation of the ^3*^C_60_ state and not the ^3*^Bodipy state (Ziessel et al., 2009). The triplet-state quantum yields of the dyad were not studied. The main photophysical processes is presented in Supplementary Figure 6.

D'Souza and Ng et al. prepared a PTZ–AzaBodip—C_60_ triad (Shi et al., 2013; Bandi et al., 2015; Collini et al., 2017). Photoexcitation of the Bodipy unit lead to the formation of PTZ^+•^−azaBodipy^−•^-C_60_ and PTZ^+•^−azaBODIPY−${\text{C}}_{60}^{-•}$ CT states. The CR lead to the formation of the ^3*^AzaBodipy state. The ISC quantum yield was not reported.

**Figure 14:**
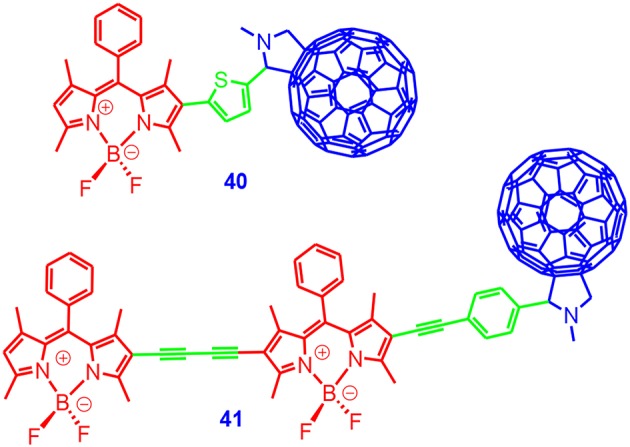
Bodipy–C_60_ dyads 40 and 41 as triplet photosensitizers.

Inspired by these studies, we prepared a Bodipy–C_60_ dyad (Figure 14), and the photophysical property was studied (Wu et al., 2012b). The absorption wavelength of the dyads can be easily changed by using different organic chromophores; **40** showed an absorption band at 515 nm, whereas **41** showed absorption at 590 nm. Note that the S_1_ state energy level of C_60_ moiety was 1.77 eV. Thus, singlet energy transfer from the Bodipy unit to the C_60_ unit is possible, although this is not a typical scenario for Förest resonance energy transfer (FRET), since the S_0_ → S_1_ transition of the C_60_ unit is very weak (Lakowicz, 1999).

The fluorescence of the Bodipy units in the dyads was strongly quenched, indicating either singlet energy transfer or electron transfer from the Bodipy units to the C_60_ unit. With nanosecond transient absorption spectroscopy, an excited state absorption band centered at 720 nm was observed, which was the characteristic absorption of the ^3^${\text{C}}_{60}^{*}$ state. The triplet-state lifetime was determined as 33.3 μs (Wu et al., 2012b). Since the dyads showed strong absorption in the visible spectral region and long-lived triplet excited states, we used the dyads for TTA upconversion. The upconversion quantum yields were up to 2.9% (**40**) and 7.0% (**41**). We determined the singlet oxygen quantum yield of an analog of **40** as 81%, thus indicating the ISC of the C_60_-Bodipy dyads was efficient (Huang et al., 2013a). To the best of our knowledge, this was the first application of C_60_-organic chromophore dyads for TTA upconversion. Following this line, we prepared a C_60_-Bodipy–styrylBodipy triad, which showed broadband absorption in the visible spectral region, to enhance the photocatalytic efficiency with a white light source (Huang et al., 2013a). We also prepared C_60_-dyads containing the chromophore of perylenebisimide (Liu and Zhao, 2012), styrylBodipy (Huang et al., 2012b), and ethyne-linked Bodipy (Huang et al., 2012a; Yang et al., 2012); an efficient ISC was observed for the dyads. We proposed that these C_60_-organic chromophore dyads that showed strong absorption of visible light and a long-lived triplet state are promising triplet photosensitizers for PDT, photocatalysis, and photon upconversion.

## Radical Enhanced ISC

It has been known that the fluorescence of organic chromophores can be quenched by stable radicals (Likhtenstein et al., 2007; Li et al., 2010; Yapici et al., 2012). With TREPR spectroscopy, it was shown that there existed a spin–spin interaction between the persistent radical and the chromophore (Corvaja et al., 1995; Ishii et al., 1996, 1999, 2001; Likhtenstein et al., 2007; Dyar et al., 2015). The overall spin of the dyad may facilitate the ISC of the chromophore (Dyar et al., 2015). However, this property was rarely used for designing visible light-harvesting triplet photosensitizers. One critical issue was to fine-tune the spin–spin interaction magnitude between the radical and the chromophore in order to attain efficient ISC and a long-lived triplet state at the same time. Strong spin–spin interactions will quench the triplet state of the chromophore (Dyar et al., 2015).

**Figure 15:**
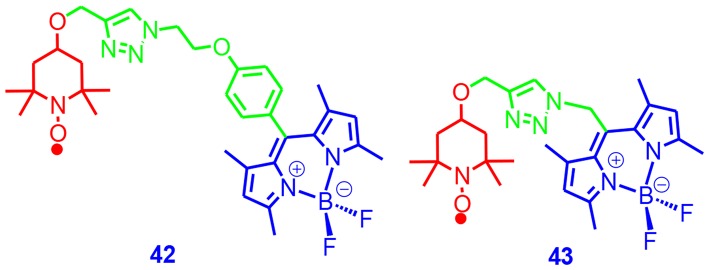
Bodipy-TEMPO dyads showing radical enhanced ISC.

We prepared two Bodipy–TEMPO dyads (Figure 15), which contain different linkers (Wang et al., 2017). The purpose of varying the linker was to tune the electron spin–spin interaction in the dyads to achieve the goal to have efficient ISC and also a long-lived triplet state. The results showed that the fluorescence of the Bodipy unit was quenched in the dyads; the fluorescence quantum yield of **42** was 29%, and the fluorescence of **43** was 5%. With nanosecond transient absorption spectroscopy, we confirmed the ISC and the formation of the ^3*^Bodipy state upon photoexcitation. The triplet-state lifetimes of the dyads were 190 and 62 μs, respectively. The singlet oxygen quantum yields of the dyads were determined as 14 and 56% for **42** and **43**, respectively. These results show that the linker length in **43** is optimal to achieve both efficient ISC and a long-lived triplet state. With TREPR spectroscopy, we observed the quartet state in a frozen solution at 80 K, indicating that the spin–spin interaction between the radical and the chromophore. In a fluid solution at 185 K, we observed the electron spin polarization of the TEMPO changed from absorption to emission with a longer delay time after the laser flash. The initial absorptive signal was due to the radical-triplet pair mechanism having a doublet precursor, and the later emissive signal was due to RTPM having a triplet precursor. The Bodipy–TEMPO dyads were used for TTA upconversion, and the upconversion quantum yield for **43** was 6.7%, but it was much lower for **42** (0.2%). The radical enhanced ISC mechanism is presented in Supplementary Figure 7.

## Conclusion

In summary, in recent years, varieties of Bodipy derivatives have been reported as having an intersystem crossing (ISC) ability, and the applications of these compounds in photocatalysis, photodynamic therapy, and photon upconversion are promising. One of the critical photophysical properties of the triplet photosensitizers is the efficient ISC ability. In this review article, we summarized the recent development in the area of Bodipy-derived triplet photosensitizers. The compounds have been introduced based on their ISC mechanisms, which include the heavy atom effect, exciton coupling, charge recombination induced ISC, using a spin converter, and radical enhanced ISC. Some transition metal complexes containing Bodipy chromophores are also introduced. The designing rationales of the molecular structures are discussed. We believe the research on the designing of new triplet photosensitizers and the application of these novel materials in the abovementioned areas will flourish.

## Author Contributions

JZ conceived the topic of the review article and wrote most of the draft. QL took part in the writing and the discussion. KC, YD, XZ, MI, and GT wrote sections of the review and prepared the figures. All the authors made comments and suggestions for the writing of the review.

### Conflict of Interest

The authors declare that the research was conducted in the absence of any commercial or financial relationships that could be construed as a potential conflict of interest.
