# Supplementary material for: Bodipy Derivatives as Triplet Photosensitizers and the Related Intersystem Crossing Mechanisms
Source: Front Chem. 2019 Dec 12;7:821. doi: 10.3389/fchem.2019.00821 (PMC6920128; doi:10.3389/fchem.2019.00821)
Supplement: Supplementary file 1 [file Data_Sheet_1.docx]

Supplementary Material

Supplementary Figure 1. 2,6-DiiodoBodipy showing ISC (Yogo et al., 2005).


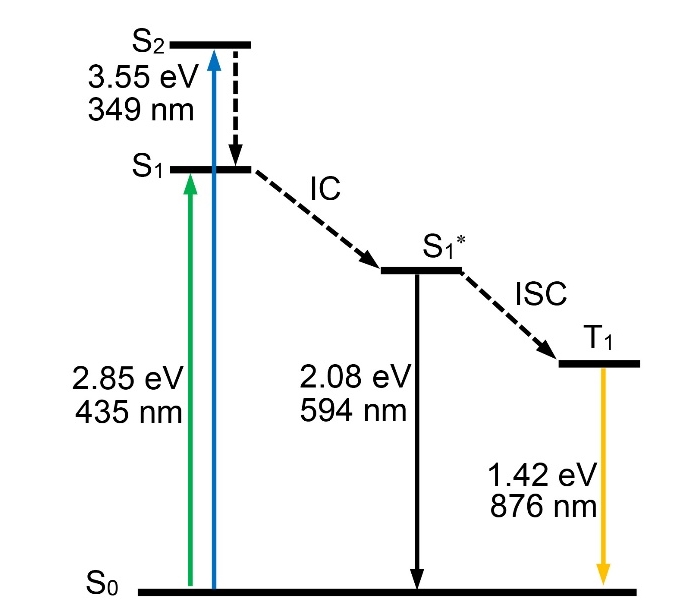


Supplementary Figure 2. The ISC mechanism of iodo-substituted Bodipy. IC denotes internal conversion. Figure reproduced from data in Nakashima et al. (2018).


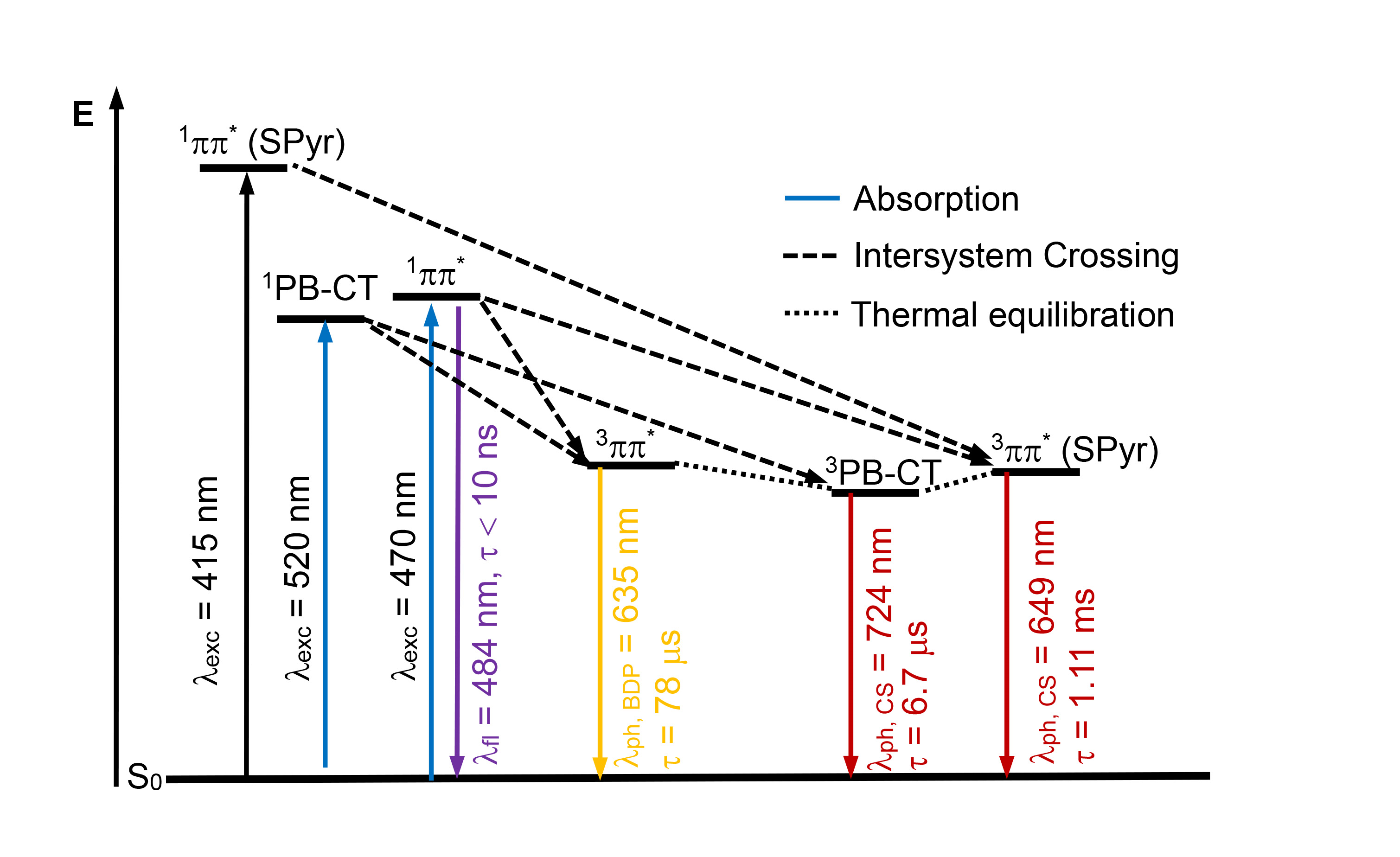


Supplementary Figure 3. The ISC mechanism of σ-Pt-BODIPY complexes. BDP: Bodipy, SPyr: mercaptopyrene, PB-CT: charge-transfer from the mercaptopyrene to BDP ligand. Figure reproduced from data in Irmler et al. (2019b).


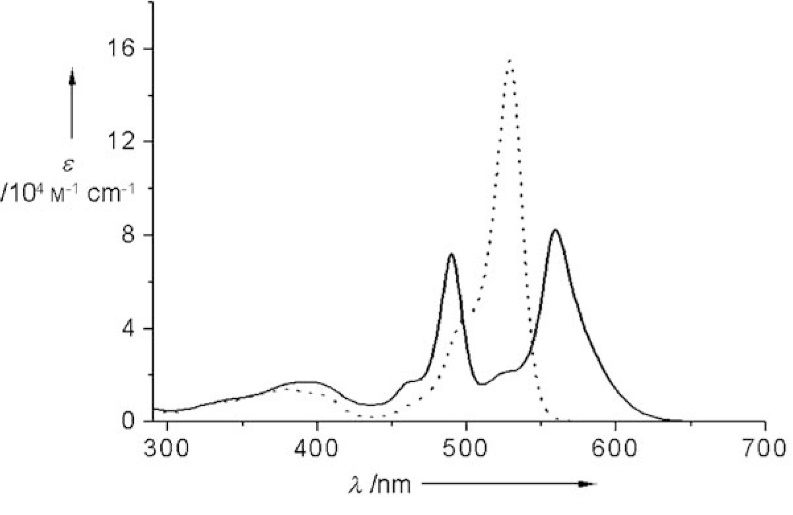


**Supplementary Figure 4.** The molecular structure and the Uv-vis absorption spectra of Bodipy dimer. Figure reproduced from data in Bröring et al. (2008)


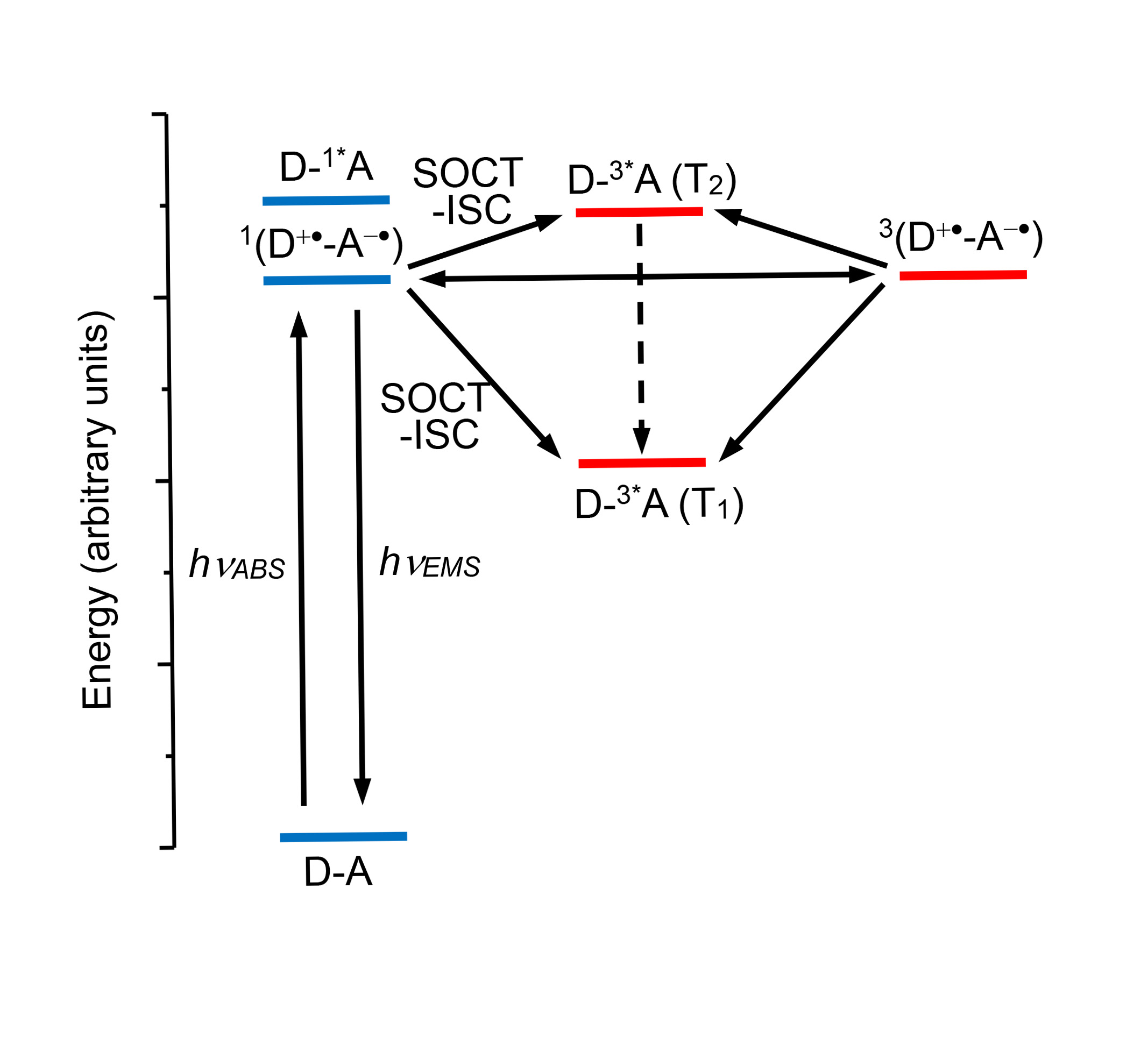


**Supplementary Figure 5.** SOCT-ISC mechanism. Figure reproduced from data in Dance et al. (2008)


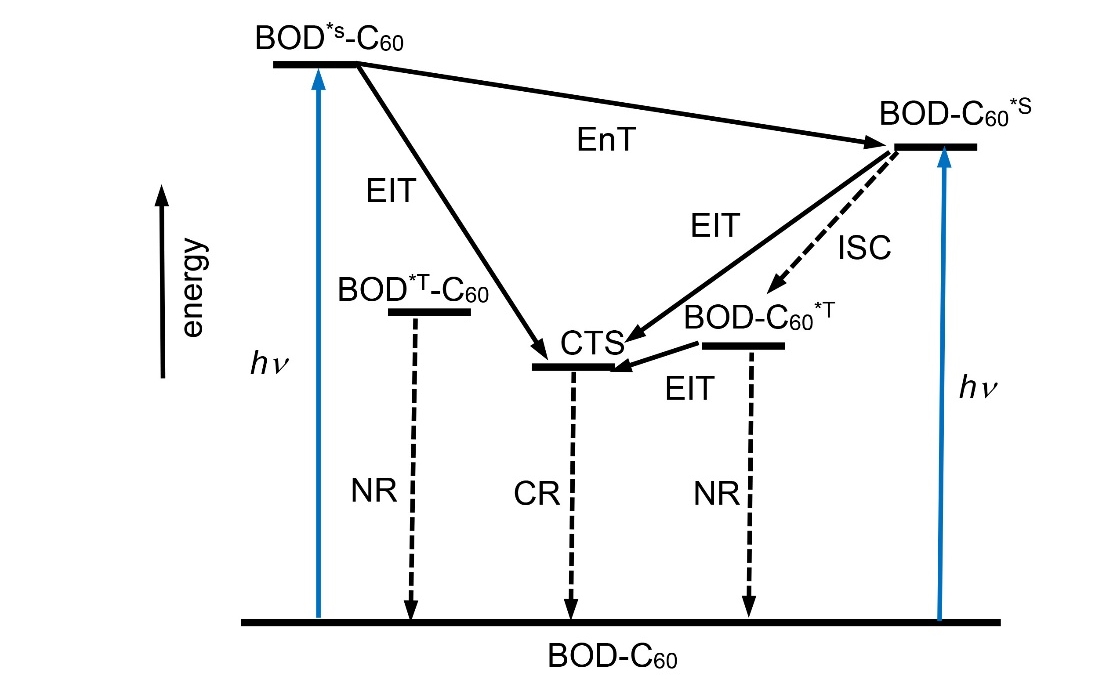


**Supplementary Figure 6.** The photophysical processes of Bodipy-C_60_ dyad derivatives. Figure reproduced from data in Ziessel et al. (2009)


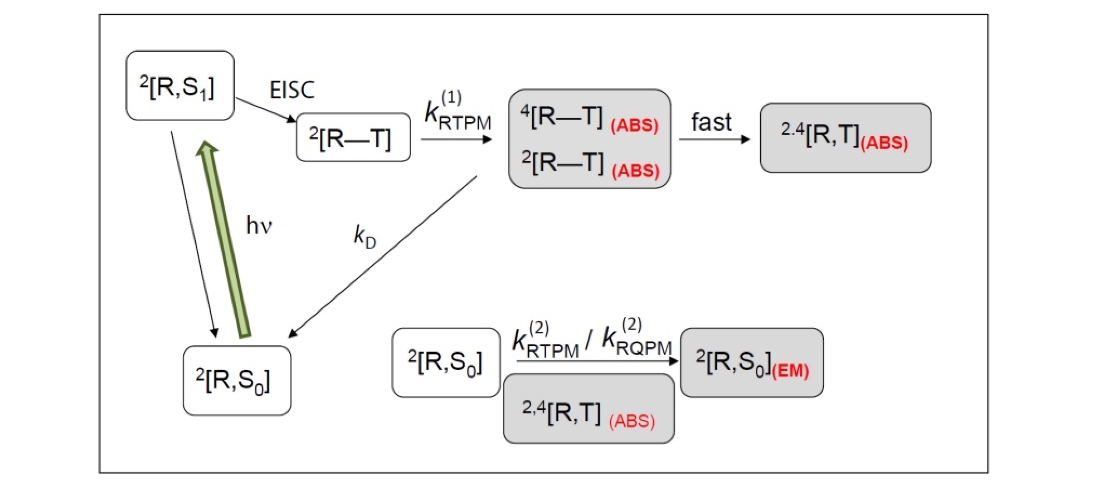


**Supplementary Figure 7.** The radical enhanced ISC mechanism of **44**. Figure reproduced from data in Wang et al. (2017)

**REFERENCES**

Yogo, T., Urano, Y., Ishitsuka, Y., Maniwa, F., and Nagano, T. (2005). Highly efficient and photostable photosensitizer based on BODIPY chromophore. *J. Am. Chem. Soc.* 127, 12162−12163. doi: 10.1021/ja0528533

Nakashima, M., Iizuka, K., Karasawa, M., Ishii, K., and Kubo, Y. (2018). Selenium-containing bodipy dyes as photosensitizers for triplet–triplet annihilation upconversion. *J. Mater. Chem. C* 6, 6208−6215. doi: 10.1039/C8TC00944A

Irmler, P., Gogesch, F.S., Mang, A., Bodensteiner, M., Larsen, C.B., Wenger, O.S., et al. (2019b). Directing energy transfer in Pt(bodipy)(mercaptopyrene) dyads. *Dalton Trans.* doi: 10.1039/C9DT01737B

Bröring, M., Krüger, R., Link, S., Kleeberg, C., Köhler, S., Xie, X., et al. (2008). Bis(BF_2_)-2,2′-bidipyrrins (bisBODIPYs): highly fluorescent BODIPY dimers with large stokes shifts. *Chem. Eur. J.* 14, 2976−2983. doi: 10.1002/chem.200701912

Dance, Z.E.X., Mickley, S.M., Wilson, T.M., Ricks, A.B., Scott, A.M., Ratner, M.A., et al. (2008). Intersystem crossing mediated by photoinduced intramolecular charge transfer:  julolidine−anthracene molecules with perpendicular π systems. *J. Phys. Chem. A* 112, 4194−4201. doi: 10.1021/jp800561g

Ziessel, R., Allen, B.D., Rewinska, D.B., and Harriman, A. (2009). Selective triplet-State formation during charge recombination in a fullerene/Bodipy molecular dyad (Bodipy=borondipyrromethene). *Chem. Eur. J.* 15, 7382−7393. doi: 10.1002/chem.200900440

Wang, Z., Zhao, J., Barbon, A., Toffoletti, A., Liu, Y., An, Y., et al. (2017). Radical-enhanced intersystem crossing in new bodipy derivatives and application for efficient triplet-triplet annihilation upconversion. *J. Am. Chem. Soc.* 139, 7831−7842. doi: 10.1021/jacs.7b02063
